# Earth’s geodynamic evolution constrained by ^182^W in Archean seawater

**DOI:** 10.1038/s41467-022-30423-3

**Published:** 2022-05-16

**Authors:** A. Mundl-Petermeier, S. Viehmann, J. Tusch, M. Bau, F. Kurzweil, C. Münker

**Affiliations:** 1 Department of Lithospheric Research, University of Vienna, Vienna, Austria; 2 Institute of Geology and Mineralogy, University of Cologne, Cologne, Germany; 3 Department of Physics and Earth Sciences, Jacobs University Bremen, Bremen, Germany

**Keywords:** Geochemistry, Sedimentology

## Abstract

Radiogenic isotope systems are important geochemical tools to unravel geodynamic processes on Earth. Applied to ancient marine chemical sediments such as banded iron formations, the short-lived ^182^Hf-^182^W isotope system can serve as key instrument to decipher Earth’s geodynamic evolution. Here we show high-precision ^182^W isotope data of the 2.7 Ga old banded iron formation from the Temagami Greenstone Belt, NE Canada, that reveal distinct ^182^W differences in alternating Si-rich (7.9 ppm enrichment) and Fe-rich (5.3 ppm enrichment) bands reflecting variable flux of W from continental and hydrothermal mantle sources into ambient seawater, respectively. Greater ^182^W excesses in Si-rich layers relative to associated shales (5.9 ppm enrichment), representing regional upper continental crust composition, suggest that the Si-rich bands record the global rather than the local seawater ^182^W signature. The distinct intra-band differences highlight the potential of ^182^W isotope signatures in banded iron formations to simultaneously track the evolution of crust and upper mantle through deep time.

## Abstract (web summary)

Banded iron formations, precipitates of Precambrian seawater, record global ^182^W isotope signatures derived from continental weathering and hydrothermal mantle fluxes into ancient oceans, tracking Earth’s geodynamic evolution through deep time.

## Introduction

The evolution of our planet and its geodynamic processes have been topics of intense debates for decades. In the past years, the now extinct ^182^Hf-^182^W radioactive isotope system with a half-life of only 8.9 Ma^1^ was able to shed new light onto early Earth evolution and deep mantle processes^2^. Our current understanding of late accretion, which is the addition of the last ~0.5% of Earth’s mass by approximately 3.9 Ga ago^3^ and convective homogenization of Earth’s mantle, the long-term preservation of early fractionated silicate reservoirs, as well as the proposed discovery of evidence for core-mantle interaction, are some processes that have benefited greatly from the study of short-lived radiogenic isotope systems^4–9^. Variations in the radiogenic ^182^W composition of rock samples can originate from (1) ancient fractionation of the lithophile element Hf and the moderately siderophile element W and/or (2) the higher incompatibility of W versus Hf in silicate systems. Thus, the contrasting partitioning of these two elements during early Earth differentiation into a metallic core and a silicate mantle, resulted in contrasting ^182^W compositions of these two individual reservoirs^10^. While most of W was sequestered into the metallic core, Earth’s silicate mantle evolved a high Hf/W ratio resulting in distinctly higher ^182^W/^184^W until ^182^Hf went extinct. In addition, differentiation processes in the early silicate Earth, such as the crystallization of an early magma ocean or the formation of very early crust, may have created reservoirs that evolved to variable ^182^W/^184^W compositions^4^. Today’s bulk silicate Earth (BSE) W isotope composition is defined as zero (µ^182^W = 0 ± 3.5, where the µ-notation reflects the parts per million deviation of the ^182^W/^184^W ratio of a sample or in this case, BSE, from that of terrestrial standards) and is interpreted to result from the decay of ^182^Hf until extinction plus the addition of late accreted material (LAM). The late accretion hypothesis has been postulated to explain the relative and absolute abundances of highly siderophile elements in the BSE by the addition of LAM in the form of chondritic meteoritic components suggested to have a ~190 ppm lower ^182^W/^184^W ratio than the present BSE^10^. This led previous studies to suggest that the mostly positive offsets of µ^182^W ~ +15 in early Earth rocks and the disappearance of ^182^W anomalies by the end of the Archean (2.5 Ga ago; Supplementary Fig. 1) mirrors the preservation of mantle reservoirs that remained unequilibrated with LAM^5^, most likely due to the absence of whole mantle convection until the onset of modern plate tectonics around 3 Ga ago^11^. Recent detailed studies suggest this progressive mantle mixing may be regionally different, reflected in a prolonged preservation of positive µ^182^W anomalies in continental crust and the progressive decrease of ^182^W anomalies in mantle-derived rocks of different cratons^11–13^. The exact timing of complete homogenization of Earth’s mantle with regard to radiogenic W isotopes, however, remains unknown. Further, our current knowledge about the µ^182^W isotope evolution of the Earth and the interpretation of the geodynamic processes are based on the fragmentary record of Archean crust that survived more than 2.5 Ga of geological processes. A prior study attempted to provide a bigger picture by studying the temporal evolution of the average upper continental crust’s (UCC) µ^182^W composition in glacial diamictites^14^. However, because of the spatial limitation of a glacier’s sampling area and a lack of global sample distribution, the recorded signatures only provide a regional image.

Elements dissolved in seawater originate from both continental surface weathering and high-temperature mantle derived hydrothermal fluids. Hence, precipitates from seawater incorporating those elements should directly reflect its isotopic composition. Banded iron formations (BIFs) are Precambrian marine chemical sediments with alternating Fe- (20–40% Fe) and Si-rich (40–50% SiO_2_) bands. BIFs are typically divided into Superior-type BIFs, i.e., deposits of large lateral extent associated with epiclastic and carbonate sediments that formed in continental shelf and slope environments of tectonically stable cratons, and Algoma-type BIFs, i.e., deposits in greenstone belts of local extent and in close association with volcanic rocks^15^. The exact depositional mechanism of BIFs is uncertain, but abiotic and biotic precipitation in a stratified, Si-rich Archean ocean^16,17^ have been invoked. The currently favored depositional mechanisms for BIFs involve passive (oxidation of uppermost water masses via oxygenic photosynthesis) and active oxidation (via anoxygenic phototrophs) of Fe^2+^ to Fe(III) (oxyhydr)oxides by microbial life. In fact, seasonal flux of Fe^2+^-rich hydrothermal plumes or oceanic bottom waters into the BIF depositional area^18^ are considered the responsible mechanism for the prominent nano- to mesoband layering in BIFs potentially reflecting diurnal to annual cycles^19^ and switches in microbial activity during warm and cold periods^20^. In contrast, other studies favor BIF mineralization from sinking authigenic precursor minerals such as iron silicates (e.g., greenalite, green rust) in the water column in which Fe(III) (oxyhydr)oxides directly precipitate from the water column and adsorb or incorporate ambient silica^17,21^. Other studies infer a development of BIF layers during diagenetic mineral phase separation from an initially geochemically homogenous Fe-Si ooze^22,23^. Despite their extensively debated depositional mechanism, BIFs have reliably shown their unique potential as geochemical archives of Precambrian seawater to reconstruct the co-evolution of landmasses, oceans and atmosphere^16,17,24–26^.

In contrast to many particle-reactive trace elements (e.g., rare earth elements, REE), W behaves conservatively in modern seawater with a suggested residence time between 14 ka and 61 ka^27,28^, which is significantly longer than the global ocean mixing time of ca. 1500 years^29^. Hence, W concentrations in the modern oceans are interpreted to be distributed homogeneously. Little is known about the concentration and behavior of W in Archean oceans. In modern seawater, the major sinks for W are Mn-oxides and Fe(III) (oxyhydr)oxides^30^. Considering significantly lower atmospheric and hydrospheric oxygen levels in the Archean, the absence of Mn-oxides as main scavengers of W suggests a similar or even longer residence time of W in Archean oceans^31^. Thus, the conservative behavior of W provides a unique opportunity to study the average W isotope composition of global seawater and with that, the µ^182^W composition of the W flux into ancient oceans from various sources. Although the exact incorporation mechanism of W into BIFs is currently unknown, the µ^182^W isotope composition of chemical sediments precipitated from seawater, such as BIFs, should directly reflect the average µ^182^W isotope composition of the W flux into ambient seawater from chemical surface weathering or submarine hydrothermal venting at the time of precipitation. Hence, the study of W isotopes in Precambrian BIFs may provide a strong chronological constraint on the nature of emerged early continents available to chemical surface weathering, as well as the upper mantle composition directly probed by input from submarine, hydrothermal vents. Thus, W isotope data obtained from BIFs may provide a global picture of the coupled geodynamic evolution of Earth’s mantle and continents through deep time.

Here, we provide an attempt to track the µ^182^W composition of Earth’s mantle and continents 2.7 Ga ago by analyzing chemical sediments that precipitated from Late Archean seawater. We discuss the ^182^W isotope data from individual Si-rich and Fe-rich layers of the Temagami BIF and their implications for the geodynamic evolution of Earth.

## Results and discussion

### Distinct ^182^W compositions of individual (meta)chert and magnetite layers in the Temagami BIF

Here, we report high-precision ^182^W isotope data for individual (meta)chert (Si-rich) and magnetite (Fe-rich) layers, and two magnetite-chert layer composites of the ~2.7 Ga old, well-preserved lower greenschist-facies Algoma-type Temagami BIF, sampled in the Temagami Greenstone Belt, Ontario, Canada. Additionally, we report ^182^W isotope data obtained from two stratigraphically associated, conformably underlying turbiditic shales, which are the closest representatives of the average UCC in the Temagami region available for chemical surface weathering. Further, we have analyzed three bulk BIF reference materials (FeR-2, ~2.7 Ga, Griffith Mine, Bruce Lake, Canada; FeR-4, ~2.7 Ga, Sherman Mine, Temagami, Canada; IF-G, ~3.7 Ga, Isua, Greenland). Importantly, we provide µ^182^W isotope data obtained by two separate instruments, a Thermal Ionization Mass Spectrometer (TIMS) and a Multi-Collector Inductively Coupled Plasma Mass Spectrometer (MC-ICP-MS) to rule out any instrumental bias. As previously demonstrated^32^, a mass-independent isotope fractionation effect on ^183^W can create analytical artifacts on measured ^182^W abundances, once mass 183 is employed for instrumental mass bias correction (^e.g.,186^,W/^183^W) when using MC-ICP-MS. Notably, both datasets (TIMS and MC-ICP-MS) reveal no resolvable difference in μ^182^W values that employed ^186^W/^183^W or ^186^W/^184^W for mass bias correction. More important^182^,W isotope compositions obtained by TIMS and MC-ICP-MS are indistinguishable. Sample details, including a comprehensive geologic background, major and trace element concentrations and Nd-Hf isotope compositions have previously been discussed elsewhere^24,25^ and are summarized in the supplementary material section. Notably, based on major and trace element systematics, the studied individual magnetite and (meta)chert layers of the Temagami BIF sampled from the roadcut at Highway 11 do not contain significant amounts of detrital components and, in marked difference to Temagami samples from the vicinity of the Sherman Mine, do not show signs of post-depositional overprint, such as fluid-rock interactions during metamorphic or mineralizing events^24,25^. Hence, they can be interpreted to reflect the pristine isotope composition of Temagami seawater 2.7 Ga ago (refer to the Methods section, as well as Supplementary Fig. 4 and the text in the Supplementary Material for further discussion).

**Fig. 1:**
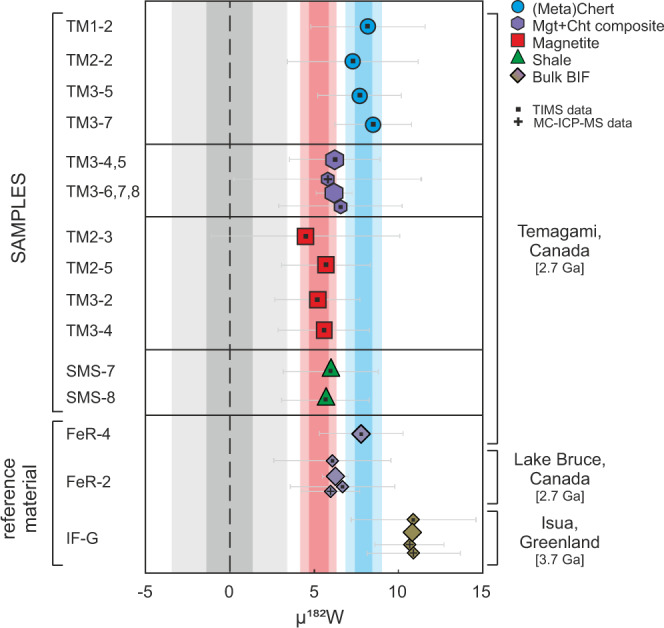
µ^182^W data for individual (meta)chert and magnetite layers and composite samples of the Temagami banded iron formation (BIF), shales and BIF reference material. The vertical light and dark gray, red, and blue bars represent the 2-standard error and 2-standard deviation, respectively, of all analyzed *Alfa Aeasar* standard solution session averages (*n* = 10), magnetite (*n* = 4), and (meta)chert samples (*n* = 4), respectively. Error bars reflect uncertainties of individual measurements (TIMS data, 2SE) or session averages (MC-ICP-MS data, 95% CI). Where applicable, small symbols represent replicate and/or duplicate measurements and larger symbols their respective averages. Symbols with dots and plus signs are data obtained by Thermal Ionization Mass Spectrometry (TIMS) and Multi-Collector Inductively Coupled Plasma Mass Spectrometry (MC-ICP-MS), respectively.

Our µ^182^W measurements of the two shales (+6.0 ± 2.8 and +5.7 ± 2.6, respectively) suggest that the average local UCC in the Temagami region exhibited a small positive µ^182^W excess at 2.7 Ga (Table 1). These values are slightly lower than those of Neoarchean shales (+7.1–+7.6) from the Pilbara craton, which represent the regional UCC composition at ~3.2 Ga and ~2.8 Ga, respectively^11^. Most crustal and mantle-derived Archean rocks analyzed for ^182^W isotope compositions display positive anomalies with µ^182^W values in the range of +12. The only exception is the southern African region where negative anomalies with µ^182^W values of down to −13 have been measured for rocks 3.0 Ga and older (Supplementary Fig. 1). The individually analyzed Temagami (meta)chert and magnetite layers show small but distinct differences in µ^182^W isotope compositions (Table 1, Fig. 1). The µ^182^W of the (meta)chert bands average at +7.9 ± 1.1 (2 SD, *n* = 4) while the average µ^182^W of the magnetite bands is distinctly lower with +5.3 ± 1.1 (2 SD, *n* = 4). This difference in ^182^W isotope composition between (meta)chert and magnetite layers corroborates evidence from Y/Ho and Eu/Sm ratios^33–35^ that the characteristic Si-rich and Fe-rich banding of BIFs represents a primary feature and cannot be the result of post-depositional processes, such as segregation from an initially geochemically homogeneous Fe-Si ooze^22^. Previous studies^24,25,33–35^ proposed that diagenetic remobilization of REY (rare earth elements and yttrium) in BIFs is rather unlikely, because it is expected that Fe oxides would show lower Y/Ho ratios at similar Eu/Sm ratios than Si-phases during diagenetic REY remobilization and re-precipitation. This is inferred from the preferential sorption of Ho (and other REE) relative to Y onto Fe(III) (oxyhydr)oxides^36^ while fractionation of Eu/Sm only occurs above ca. 250 °C^37^. However, such differences between adjacent BIF bands are not observed^33^, suggesting a primary origin of the banding. This is also corroborated by significantly different Ge/Si and Th/U ratios and ^53^Cr isotope compositions between adjacent bands in the Temagami BIF^35^. The additional evidence from ^182^W isotopes presented here strongly supports the interpretation that the alternating layers must have formed from water masses tapping different chemical reservoirs.

**Table 1:** Tungsten isotope compositions of BIF samples and reference material.

sample	Location^#^	Age [Ga]	rock type	Instrument	µ^182^W _N6/3_	±	µ^182^W _N6/4_	±	µ^183^W _N6/4_	±
*BIF samples*										
TM1–2	Temagami, Canada	2.7	chert	N-TIMS	8.2	3.4	11.7	4.4	2.4	3.5
TM2–2	Temagami, Canada	2.7	chert	N-TIMS	7.3	3.9	4.0	5.3	−1.8	4.7
TM3–5	Temagami, Canada	2.7	chert	N-TIMS	7.7	2.5	4.3	3.6	−4.1	3.1
TM3–7	Temagami, Canada	2.7	chert	N-TIMS	8.5	2.3	8.2	3.2	1.3	2.9
*average (meta) chert*					7.9	0.5^+^	7.0	3.5^+^	−0.6	3.0^+^
						1.1^−^		7.0^−^		5.9^−^
TM2–3	Temagami, Canada	2.7	magnetite	N-TIMS	4.5	5.6	4.3	8.2	5.9	6.1
TM2–5	Temagami, Canada	2.7	magnetite	N-TIMS	5.7	2.6	6.5	3.3	1.9	2.8
TM3–2	Temagami, Canada	2.7	magnetite	N-TIMS	5.2	2.5	5.1	3.3	−1.1	3.0
TM3–4	Temagami, Canada	2.7	magnetite	N-TIMS	5.7	2.8	5.6	3.7	2.1	3.1
*average magnetite*					5.3	0.6^+^	5.6	0.9^+^	2.3	2.9^+^
						1.1^−^		1.8^−^		5.7^−^
TM3–4,5	Temagami, Canada	2.7	cht^1^-mgt^1^ mix	N-TIMS	6.2	2.7	6.4	3.6	1.3	3.0
TM3–6,7,8	Temagami, Canada	2.7	cht^1^-mgt^2^ mix	N-TIMS	5.8	5.6	7.0	7.6	4.2	7.5
TM3–6,7,8 dup*				MC-ICP-MS	6.6	3.7	6.5	5.6	−0.1	5.4
SMS-7	Temagami, Canada	2.7	shale	N-TIMS	6.0	2.8	6.3	3.9	2.9	3.3
SMS-8	Temagami, Canada	2.7	shale	N-TIMS	5.7	2.6	6.5	3.4	−0.4	2.8
*BIF reference material*										
FeR-2	Bruce Lake, Canada	2.7	bulk BIF	N-TIMS	6.1	3.5	2.3	4.6	−2.8	3.9
FeR-2 *rep*				N-TIMS	6.7	3.1	8.8	3.9	2.3	3.1
FeR-2 *dup*				MC-ICP-MS	6.0	1.7	5.0	1.7	−0.3	1.2
FeR-4	Temagami, Canada	2.7	bulk BIF	N-TIMS	7.8	2.5	9.9	3.4	0.6	2.7
IF-G	Isua, Greenland	3.7	bulk BIF	N-TIMS	10.9	3.7	9.7	4	−2.0	3.6
IF-G *dup*				MC-ICP-MS	10.6	2.0	10.9	2.8	1.1	2.6
IF-G *dup*				MC-ICP-MS	10.9	2.8	10.9	3.3	−1.8	2.5

rep - replicate, indicates a measurement from the same sample digestion and chemical separation.

dup - duplicate, indicates a measurement of a sample from a separate digestion and chemical separation.

dup* - duplicate from the same sample digestion, but different chemical separation.

^#^refer to ref. ^25^ for location details.

cht1-mgt1 mix and cht1-mgt2 mix indicate a mixture of (meta) chert and magnetite layers at proportions 1:1 and 1:2, respectively. Proportions represent amounts of individual layers and not the absolute mass fraction.

bulk BIF - sample consists of multiple layers of (meta)chert and magnetite.

N-TIMS - Thermal Ionization Mass Spectrometry in negative ionization mode at the University of Vienna.

MC-ICP-MS – Multi-Collector Inductively Coupled Plasma Mass Spectrometer at the University of Cologne.

µ^*i*^W - (^*i*^W/^184^W_sample_/^*i*^W/^184^W_standard_−1) × 10^6^, where *i* is 182 or 183. N_6/3_ and N_6/4_ imply the normalization to ^186^W/^183^W and ^186^W/^184^W, respectively.

For N-TIMS uncertainties represent the 2x standard error (2SE) of individual measurements, for MC-ICP-MS uncertainties represent the 95% confidence interval (95% CI) of individual measurements.

^+^ and ^−^ give the 2SE and 2x standard deviation of the average (meta) chert and magnetite layers, respectively (*n* = 4).

### Scenarios to interpret heterogeneous µ^182^W signatures in individual Temagami BIF layers

**Fig. 2:**
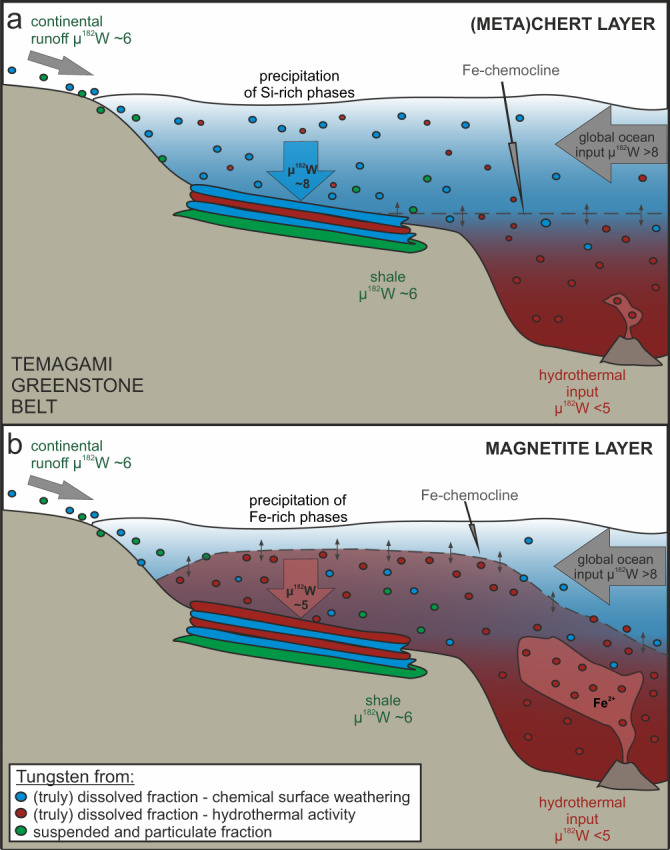
Cartoon illustrating the deposition of (a) Si-rich (meta)chert and (b) Fe-rich magnetite layers in the Temagami banded iron formation (BIF) with respective µ^182^W isotopic compositions. Filled circles illustrate W present in seawater in the suspended and particulate fractions (green) and in the (truly) dissolved fractions derived from chemical surface weathering of upper continental crust (UCC; blue) and hydrothermal activity (red). During times of high clastic sedimentation, shales are deposited with µ^182^W compositions of ~+6, representing the W isotope composition of the regional Temagami UCC (green layer). Dissolved W from both chemical surface weathering of the UCC (>+ 8) and hydrothermal activity (<+5) is transported into the seawater. The Fe-chemocline represents the interface between an upper, Fe-poor but Si-rich and a lower, ferrous iron-rich water mass in a stratified Archean ocean. **a** During periods of low hydrothermal activity, mainly Si-rich precipitates form in the upper water column (blue layer). Because of the long residence time of W in seawater, the µ^182^W composition of the (meta)chert layer represents the composition of the open, potentially global ocean, which reflects the average µ^182^W composition of the total W flux from global UCC surface weathering (>+8) plus a potential hydrothermal component of <+5. **b** In times of increased hydrothermal activity, ferrous iron-rich bottom waters are transported into the BIF depositional environment, resulting in the precipitation of Fe(III) (oxyhydr)oxides that eventually turn into magnetite (red layer). The µ^182^W composition of this magnetite layer is dominated by W from hydrothermal activity plus potentially a (smaller) UCC weathering component.

The distinct µ^182^W signatures of the Temagami (meta)chert and magnetite BIF layers alone could have multiple implications and may be explained in various ways. While shales were deposited during times of high clastic input from the continents, BIFs precipitated in periods of low clastic input, forming sets of alternating Si- and Fe-rich layers (Fig. 2). Different interpretations and scenarios for the distinct µ^182^W signatures observed in the (meta)chert and magnetite layers of the Temagami BIF are discussed in detail below:

The similarity of the µ^182^W composition between the magnetite layers (+5.3) and shales (+5.9) may suggest a common W source. The shales are interpreted to represent the composition of the Temagami regional UCC. Consequently, the W isotope composition of the magnetite layers would then indicate a source of dissolved W flux of chemically weathered regional continental crust into the seawater. In contrast, the (meta)chert layers, characterized by distinctly higher µ^182^W (+7.9), could represent the W composition of ambient seawater including submarine hydrothermal flux and thus, the minimum µ^182^W value of the upper mantle. In fact, the 2.7 Ga Boston creek komatiites located within the AGB, north of Temagami, show an average µ^182^W value of +12^38^ and are significantly higher than the shales and magnetite layers but closer to the µ^182^W composition of the cherts. However, the Boston creek komatiites have been interpreted to have derived from a deep mantle source^38^ and thus, despite its geographic proximity, its composition may not be directly comparable to the ambient upper mantle composition at 2.7 Ga, inferred from hydrothermal flux into seawater. No µ^182^W data for upper mantle-derived rocks at or around 2.7 Ga are currently available. Hence, the radiogenic W isotope composition of the upper mantle towards the end of the Archean remains ambiguous.

An alternative interpretation for the µ^182^W similarities between the shales and the magnetite layers would be a predominant incorporation of crustal material in the form of detrital components into the magnetite layer. However, trace element systematics and other geochemical proxies in both (meta)chert and magnetite layers of the Temagami BIFs strongly argue against a significant detrital contamination^24,25^ (Supplementary Material). However, the provenance and elemental distribution of the element W in the individual BIF layers and its mineral phases is currently unknown. Although highly unlikely when considering currently available geochemical evidence^24,25^, a contribution of detrital W to the µ^182^W composition of the magnetite layers cannot be excluded with certainty and still may explain the similarity between the µ^182^W compositions of the magnetite layers and shales. However, detrital contamination would then only have affected the W budget and spared other trace elements, which seems difficult to envision. Additional data and detailed studies on the behavior of W in Archean seawater and its incorporation into BIFs will be necessary to definitively prove or refute this interpretation.

Based on trace element compositions, Th/U and Ge/Si ratios as well as radiogenic Nd-Hf and stable Cr isotope systematics, previous studies that investigated individual layers from the Temagami BIF have suggested that the (meta)chert layers reflect the seawater composition that was controlled by chemical surface weathering of emerged continental crust^24,25,35^. During periods of no or only minor upwelling of ferrous iron-rich bottom waters affected by high-temperature hydrothermal input into the BIF depositional environment, Si-rich layers precipitated above the Fe-chemocline (Fig. 2a). However, the Temagami (meta)chert layers (+7.9) show distinctly higher µ^182^W compositions compared to the Temagami shales (+5.9), considered representative of the regional UCC. Consequently, this discrepancy indicates that the ^182^W isotope composition of the (meta)cherts reflects the signal of a water mass derived from the open ocean of which the W is supplied from global sources, rather than from local Temagami landmasses. This interpretation is in line with a proposed residence time of W in Archean oceans that is longer than global ocean mixing times and its presumed conservative behavior not only in modern but also in Late Archean seawater. In times of upwelling of marine bottom waters or anoxic, Fe^2+^-rich plumes into the upper portions of the ocean, the Fe-chemocline is shifted upwards towards the water level and Fe(III) (oxyhydr)oxide layers precipitate^18^, that later turn into magnetite bands (Fig. 2b). The average µ^182^W of these magnetite layers (+5.3), thus, should be close to that of the hydrothermal input which likely represents the upper mantle composition at the time of hydrothermal activity. Whether the lower ferrous iron-rich ocean layer in a chemically stratified Archean ocean was globally connected or only related to regional submarine volcanism is currently unknown. Hence, the µ^182^W signature of the magnetite layer may represent either the global or regional upper mantle composition.

In light of previously published interpretations that provide geochemical evidence for the origin of the (meta)chert and magnetite layers^24,25,35^, and considering our new ^182^W isotope data, we favor the latter scenario to explain the formation of individual Fe- and Si-rich layers of the Temagami BIF (Fig. 2). It is, however, important to note, that the (meta)chert and magnetite layers may not represent the source composition of the pure Si-rich and Fe-rich endmember, respectively (Fig. 2). For example, while the average µ^182^W composition of the (meta)cherts is considered to reflect the signature of the global flux from chemical weathering of the UCC, observed positive Eu anomalies^24^ are indicative of a high-temperature hydrothermal component in the water masses from which the Si-rich layer precipitated. Similarly, the decoupling of Hf and Nd isotopes observed in magnetite corresponds to that seen in (meta)chert layers^25^, indicating the presence of an UCC component in the magnetite layers as well. µ^182^W compositions do not correlate with W concentrations (Supplementary Fig. 2). Similar W concentrations in the studied (meta)chert (average 550 ppb)^24^ and magnetite layers (average 450 ppb)^24^ likely buffer the primary µ^182^W signature of the individual layers (Supplementary Fig. 3). Still, the µ^182^W signatures of the individual layers must be considered minimum (for the UCC) or maximum (for the upper mantle), rather than pure endmember µ^182^W compositions of their respective sources. The true µ^182^W difference between the average global UCC and upper mantle endmembers would then be greater than what is measured in individual (meta)chert and magnetite layers.

### Significance of ^182^W isotope signatures in BIFs for the geodynamic evolution of Earth

All studied Temagami BIF samples show positive ^182^W offsets up to ~8 ppm compared to the inferred modern BSE value, suggesting an average positive µ^182^W signature of the total global W flux into the ocean at 2.7 Ga. With the exception of samples from the southern African region^14,39,40^, previously studied Archean rocks, irrespective of rock type, are characterized by positive µ^182^W compositions^4,5,12,13,41^ (Supplementary Fig. 1). However, it is important to mention that the existing Archean rock database is rather biased towards distinct sampling areas, and several Archean cratons have not yet been analyzed for µ^182^W compositions. Hence, it is ambiguous whether samples from the southern African region are unique in their negative µ^182^W composition or whether negative µ^182^W in Archean rocks are more ubiquitous. Archean samples characterized by negative µ^182^W signatures may simply be under-sampled and their scarcity, therefore, is merely a result of sampling bias. If, as inferred from the results of this study, the µ^182^W composition of BIF layers reflects that of the global W flux into seawater, the µ^182^W measured in this study implies an UCC and upper mantle composition that on average is dominated by a positive µ^182^W signature at 2.7 Ga. This suggests the negative µ^182^W compositions observed in Archean samples from southern Africa to be an exception rather than reflecting sampling bias. Tungsten-182 data of rocks from individual Archean outcrops provide only information on the crust that has been preserved until today. Analyses of individual pure and pristine BIF layers, specifically of the (meta)chert layers, however, integrate the ^182^W composition of all the UCC exposed to chemical surface weathering at the time of BIF deposition. Further, the concurrent study of individual (meta)chert and magnetite layers from the same BIF allows for simultaneous tracking of the ^182^W composition of both the upper mantle and the continental crust at a certain point in time. Applying the short-lived radiogenic W isotope system to BIFs of different ages, therefore, has the unique potential to track the geodynamic evolution of the crust-mantle system through Precambrian times with regards to mantle homogenization.

In conclusion, distinct ^182^W isotope compositions of alternating (meta)chert and magnetite layers infer a primary origin of the banding in BIFs. Positive µ^182^W values of up to +7.9 ± 1.1 of the (meta)chert layers are interpreted to represent a global UCC signature. We infer the slightly lower positive anomalies in the magnetite layers (µ^182^W = +5.3 ± 1.1) to reflect that of the (regional) upper mantle. The new µ^182^W data for individual (meta)chert and magnetite layers of the Temagami BIF highlight the unique applicability of W isotopes in BIFs and potentially other marine chemical sediments as unique geochemical archives to investigate the geodynamic evolution of our planet and to better understand the nature and emergence of the earliest continents on Earth.

## Methods

### Samples

The samples analyzed in this study comprise four (meta)chert layers (TM1–2, TM2-2, TM3–5, TM3–7), four magnetite layers (TM2-3, TM2–5, TM3-2, TM3-4), two composites representing mixtures of (meta)chert and magnetite layers (TM3-4 and TM3–5; TM3–6, TM3–7 and TM3–8) from alternating (meta)chert and magnetite layers of the Temagami BIF, as well as two associated, stratigraphically conformably underlying shales (SMS-7, SMS-8) from the 2.7 Ga old Temagami Greenstone Belt, Canada. Samples macroscopically free of secondary veins were prepared by cutting individual layers with a diamond saw and subsequent powdering using an agate or ceramic mill. Composite samples TM3-4&5 and TM3–6,7&8 were crushed with a metal-free tool and powdered in a ceramic mill. In addition, the BIF reference materials FeR-2 (~2.7 Ga, Griffith Mine at Bruce Lake, Canada), FeR-4 (~2.7 Ga, Sherman Mine, Temagami, Canada), and IF-G (~3.7 Ga, Isua, Greenland) were analyzed for µ^182^W isotope composition. A more detailed sample description and geologic overview of Temagami can be found in the supplementary material.

Major and trace element concentrations and Nd-Hf isotope compositions have been reported by previous studies^24,25^ on the same (meta)chert and magnetite bands, however, from separate powders obtained from microdrill cores few centimeters away from the material processed in this study. Yet, the good data overlap between the two studies suggests a homogeneous composition of individual bands. Hence, previously determined trace element concentrations in combination with W isotope data of this study can be used to assess detrital contamination and/or post-depositional alteration effects on the studied samples. In brief, W concentrations show no correlation with almost immobile elements typically associated with detrital aluminosilicates (e.g., Zr;^34^ Supplementary Fig. 4a). Even though a weak correlation between Zr concentrations and ^182^W isotope compositions exists (*r*² = 0.3899; Supplementary Fig. 4b), this is likely the result of minor Si-rich and Fe-rich phases in the magnetite and (meta)chert layers, respectively, and not from the addition of a detrital component. This is evidenced by the correlation plotting far off the mixing curve between the average Temagami shale, the most likely representatives of local clastic material, and the (meta)chert sample with the highest ^182^W composition (Supplementary Fig. 4b). Hence, a significant contribution from a detrital component, which could have affected the ^182^W isotope signatures of the studied samples can be excluded in the individual (meta)chert and magnetite layers. Similarly, no correlations between W concentrations (*r*² = 0.0345) and/or ^182^W isotope compositions (*r*² = 0.0312) with the fluid mobile element Sr, often used to monitor post-depositional mobility of the respective elements during fluid-rock interactions, can be observed in the studied samples (Supplementary Figs. 4c and 4d). Refer to supplementary material for an extended discussion.

### Tungsten purification and mass spectrometry

University of Vienna: Tungsten concentrations have previously been reported in^24^ and based on those compositions, between one and ten grams of sample powder were digested in up to 50 ml of a mixture of HF:HNO3:HCl 1:1:2 for three days at 145 °C. A dry down was followed by re-dissolution in 8 M HCl for three days at 130 °C. The samples were subsequently dried down twice in 8 M HCl to fully convert the samples to chloride form. Tungsten was separated and purified following a three-step ion-exchange chromatography method described in^42^. The amount of step-1 cation exchange resin was adjusted to the amount of iron present in the samples requiring distinctly higher resin volumes for magnetite and composite/bulk BIF samples relative to (meta)chert samples. After the final purification step, the solution was dried down and repeatedly dried down with a mixture of HNO_3_:HCl:H_2_O_2_ 4:2:1 to remove any organic residue before analysis. Final W yields were in the range of 65–85% for all samples. Approximately 10 g of composite sample TM3–6,7,8 was digested following the protocol described above. After the last HCl dry-down step, the sample was picked up in 30 ml 8 M HCl and split into two beakers, containing 10 and 20 ml, respectively. The dried down 20 ml aliquot was then sent to the University of Cologne for W purification and subsequent analysis using a Multi-Collector ICP-MS.

Between 700 and 1000 ng of W were dried onto a pure Re filament and coated with an electron emitter consisting of 15 µg La and 5 µg Gd. Tungsten isotope compositions were measured using a Thermo Fisher^®^ Triton and Triton XT Thermal Ionization Mass Spectrometer in negative ionization mode (N-TIMS) at the Department of Lithospheric Research, University of Vienna following a modified method described in^43^. The measurements comprise two acquisition lines with 34 s integration and 10 s idle time and rotating amplifiers. A 360 s baseline measurement was performed before every block each consisting of 20 cycles. Source focus and peak centers were done before every second block. ^186^W^16^O_2_^18^O and ^187^Re^16^O_2_^18^O were measured with every run to perform per-integration oxide interference corrections using amplifiers equipped with 10^12^ Ω (Triton) and 10^13^ Ω (Triton XT) resistors. Isotope ratios were corrected for instrumental mass bias by normalizing to ^186^W/^183^W = 0.92767 or ^186^W/^184^W = 1.98594^44^. All data are reported as µ^182^W and µ^183^W, which are the deviations of ^182^W/^184^W and ^183^W/^184^W, respectively, of a given sample from that of repeated measurements of the Vienna *Alfa Aesar* laboratory W standard solution within a sample campaign (Supplementary Data 1). The average µ^183^W of all samples is 0.8 ± 1.3 (2SE, *n* = 16) and thus, identical within uncertainties to the average *Alfa Aesar* W standard data (Table 1) ruling out potential nuclear field shift issues.

University of Cologne: The separation of W followed established protocols, which were previously described in more detail^11,40^. In short, up to ca. 6 g sample powder was fully digested and aliquots equivalent to 1 g sample material were loaded onto ion-exchange columns. The chemical purification of W for high-precision isotope composition analysis was achieved by a four-stage ion-exchange chromatography procedure employing cation (AG 50 W-X8 resin, column I), anion (AG 1-X8 resin, column II), TEVA (column III) and TODGA resin (column IV). The final W-bearing eluate was directly loaded onto BioRad Poly-Prep® columns filled with ≥0.8 ml Eichrom prefilter® material to extract organic compounds. This, together with a threefold treatment with 80 μl of conc. HNO_3 _— 30% H_2_O_2_ at max. 60 °C after all dry-down steps during and after the chromatographic separation fully removed mass-independent effects on ^183^W^41^. The total procedural yields of W were in the range of 69–95%.

The high-precision W isotope composition measurements at University of Cologne were conducted on a Thermo Fisher^®^ Neptune Plus MC-ICP-MS and mainly followed established protocols, which are described elsewhere^11,41^. In short, samples were measured at intensities ranging from ca. 11 to 21 V for ^182^W (using 10^11^ Ohm amplifiers) at an uptake rate of ca. 60 μl/min either using an Aridus II (Teledyne CETAC) or an Apex Omega (Elemental Scientific) desolvating system. The correction for mass dependent isotope fractionation followed the exponential law and either involved ^186^W/^183^W = 0.92767 or ^186^W/^184^W = 1.98594^44^ for normalization. Samples were always bracketed by a concentration-matched certified reference material (NIST SRM 3163) to report relative W isotope compositions in the μ notation (equivalent to ppm). All samples were repeatedly analyzed (*n* = 7–14) and uncertainties for average W isotope compositions are correspondingly reported as 95% confidence intervals.

In every analytical session at least one Cologne in-house rock reference material was analyzed (*LP 1*, *AGC 351*, *160245*) that, together with the samples, always was passed individually through the separation protocol. In-house rock reference material *LP 1* is a historical OIB from La Palma (erupted 1480), *AGC 351* is a 3455 Ma old granitic gneiss from the Kaapvaal Craton, Southern Africa^45^ and *160245* is a 3270 Ma old Komatiite from the Pilbara Craton, NW Australia^11^. The W isotope composition for Cologne in-house rock reference materials reported in this study overlaps with the long-term average that has previously been obtained to assess the intermediate precision for W isotope composition analysis at University of Cologne (Supplementary Data 2)^11,40,41^.

## Supplementary information

Supplementary Information

Description of Additional Supplementary Files

Supplementary Data 1

Supplementary Data 2

## Data Availability

All data that support the findings of this study are available in the main manuscript and supplementary material.
