# Supplementary material for: Earth’s geodynamic evolution constrained by 182W in Archean seawater
Source: Nat Commun. 2022 May 16;13:2701. doi: 10.1038/s41467-022-30423-3 (PMC9110358; doi:10.1038/s41467-022-30423-3)
Supplement: Supplementary file 1 — Supplementary Information [file 41467_2022_30423_MOESM1_ESM.pdf]

Supplementary material for

Earth's geodynamic evolution constrained by ^{182}W in Archean seawater

A. Mundl-Petermeier, S. Viehmann, J. Tusch, M. Bau, F. Kurzweil, C. Münker

Supplementary figures:

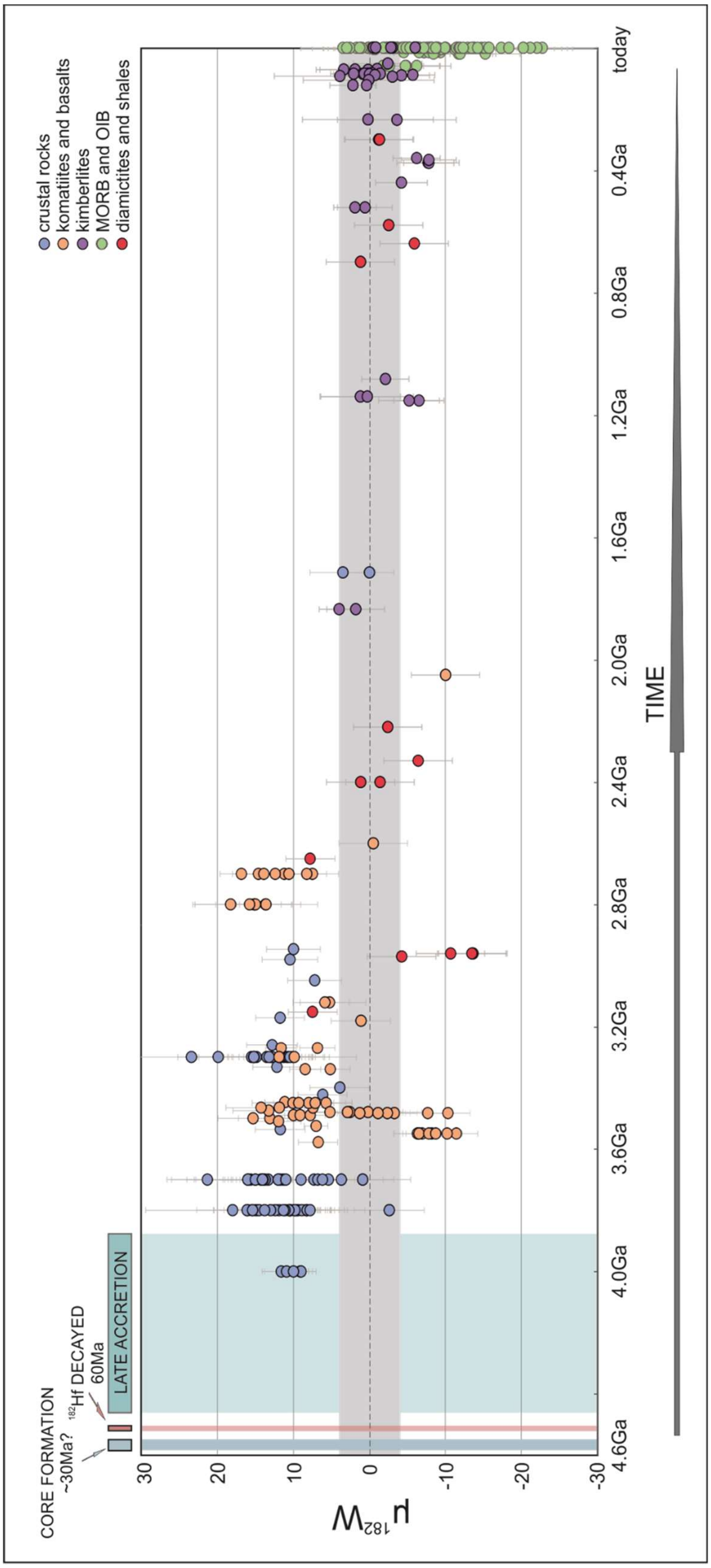

***Supplementary Figure 1.* Compilation of previously published μ^{182}W isotope data.** Most of the Archean (4.0 – 2.5 Ga) rocks are characterized by positive μ^{182}W. The only Archean samples with negative μ^{182}W are found in komatiites and glacial diamictites from southern Africa. After the Archean, rocks with positive μ^{182}W vanish and all post-Archean samples are characterized by μ^{182}W = 0 or <0. Differently colored symbols discriminate different rock types. Data shown are from[1–26].

Supplementary Figure 2. **Tungsten isotope composition versus W concentrations in chert and magnetite layers, as well as one shale (SMS-8) and one reference bulk BIF (FeR-4) from the Temagami Greenstone Belt.** A correlation between $\mu^{182}W$ and W concentrations (from[27]) cannot be observed.

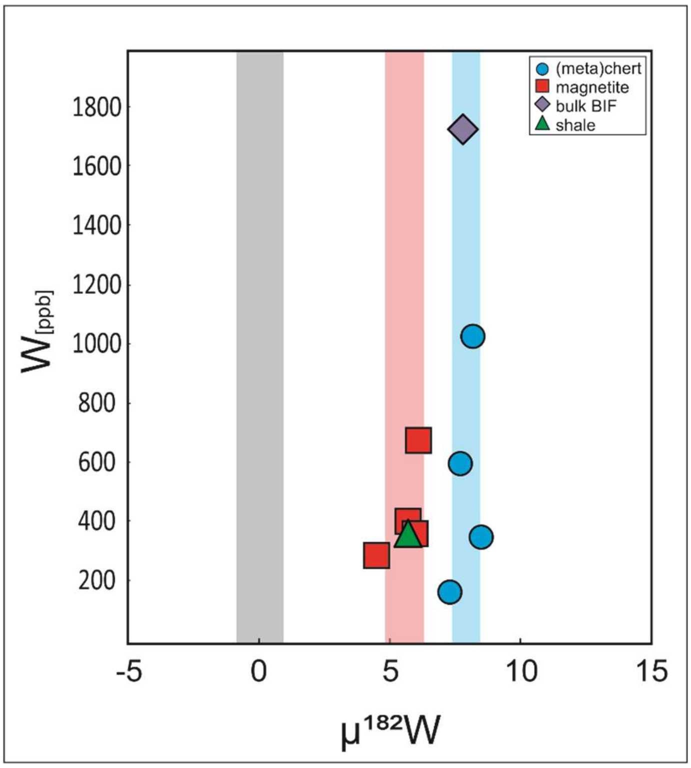

Supplementary Figure 3. **Effect of mixing between chert and magnetite component on the $\mu^{182}W$ composition.** The red square and blue filled circle represent the average $\mu^{182}W$ composition of all analyzed magnetite (n=4) and chert (n=4) samples, respectively. Multi-colored symbols show the effect of mixing between a magnetite and chert component at 5 % increments using the average $\mu^{182}W$ composition and concentrations of the chert ($\mu^{182}W$ = +7.9; 550 ppb W) and magnetite ($\mu^{182}W$ = +5.6; 450 ppb W) layers analyzed in this study and from[27]. This mixing model shows that large amounts of inter-component contamination would be required to significantly affect the ^{182}W isotopic composition.

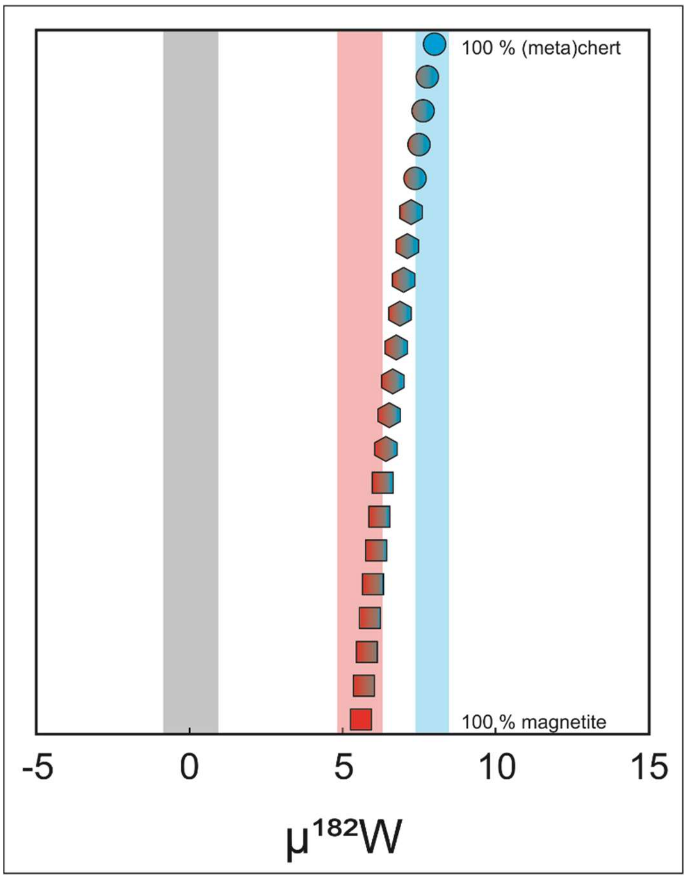

Supplementary Figure 4. Tungsten concentrations and ^{182}W isotope compositions versus Sr and Zr concentrations to determine detrital contamination and/or post-depositional alteration effects on the studied samples. a) No correlation of Zr and W concentrations indicative of a significant detrital contamination can be observed in the studied samples. b) The weak correlation between Zr concentrations and $\mu^{182}W$ isotope compositions does not fall on the mixing curve between the average shales and the chert sample with the highest $\mu^{182}W$ and thus, likely results from minor magnetite-chert source component mixing. The "x"-s represent 5 % mixing increments. c and d) Based on a lacking correlation between Sr and W concentrations as well as $\mu^{182}W$ isotope compositions, relevant post-depositional alteration can be excluded. Tungsten, Zr and Sr concentrations from[27].

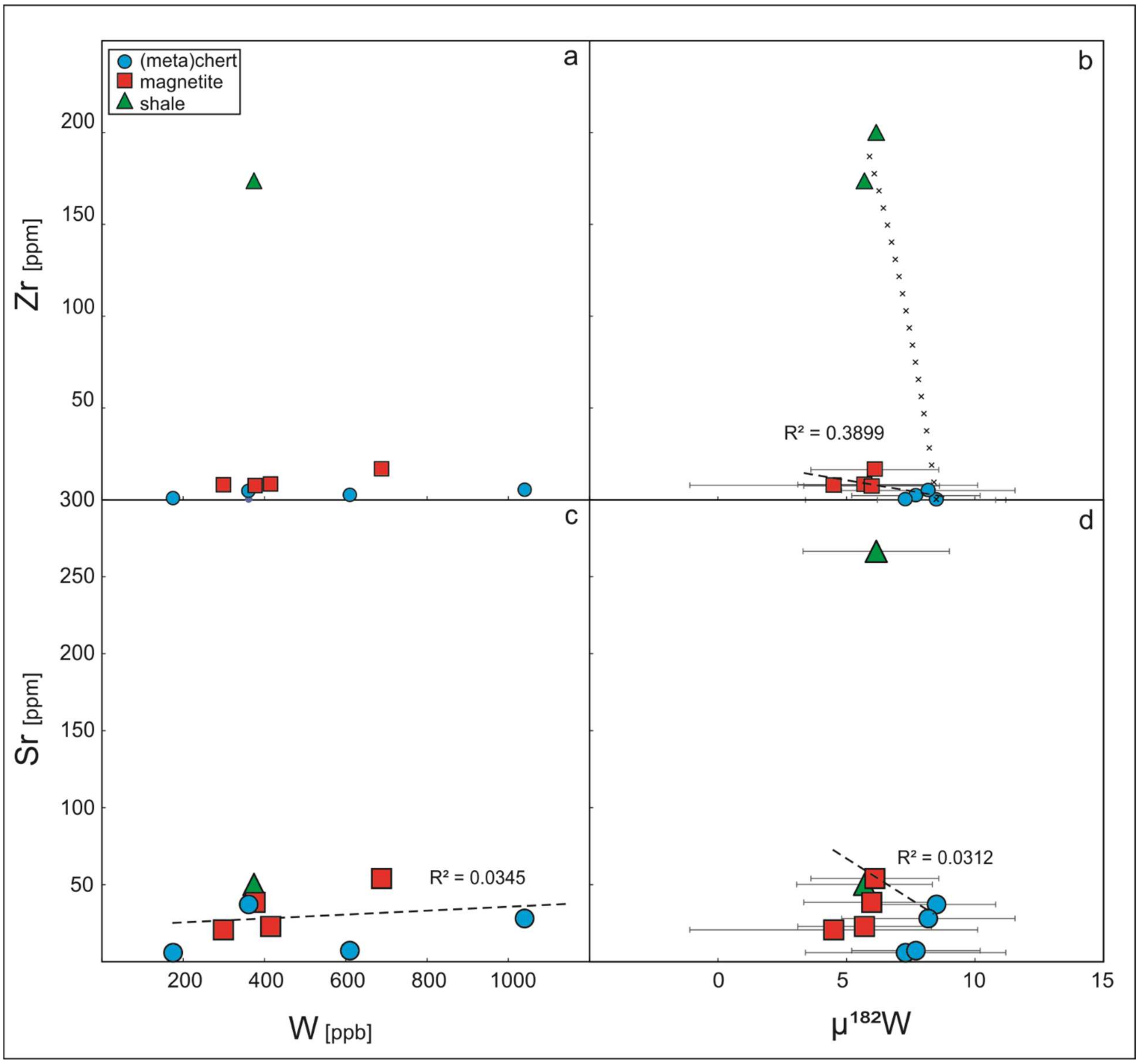

Supplementary text:

Regional geology & sample selection

The Temagami Banded Iron formation (BIF) is located in the Temagami Greenstone Belt that is part of the Neoarchean Abitibi Greenstone succession in the Canadian Shield. The up to 200 m thick Algoma-type BIF in the Temagami area crops out in a syncline and is associated with (meta)sedimentary and (meta)volcanic units (Supplementary Figure 5a). The here studied BIF samples originate from a road cut on Highway 11 north of the village of Temagami (47°04‘24’’ N; 79°47’31’’ W) and encounter the BIF succession in the southern fold limb. The BIF at this sampling location consists of well-preserved alternating (meta)chert and magnetite mesobands (Supplementary Figure 5b) and experienced only lower greenschist facies metamorphism[28]. In contrast, the reference BIF material FeR-4 from the southern pit of the Sherman Mine at Temagami show secondary hematite enrichment in (meta)chert layers and macroscopic visible sulfide grains (e.g.,[29]) that are not observed in the here studied Temagami BIF samples. The depositional age of the Temagami BIF is bracketed by U-Pb zircon ages of 2736 ± 3 Ma from underlying metavolcanics and 2687 ± 2 Ma of a crosscutting rhyolitic dyke[30]. Both Fe- and Si-rich layers from the same hand specimens used in this study show no geochemical evidence for detrital aluminosilicate contamination on the rare earth element and Hf-Nd isotope budgets[27,31]. These layers were directly dated via the radiogenic Sm-Nd and Lu-Hf isotopes and yield 2605 ± 140 Ma and 2760 ± 120 Ma[31], respectively, overlapping with the proposed depositional age and suggesting a negligible impact of post-depositional overprints on the Hf-Nd isotope systems. Thus, despite their Neoarchean age, our samples from the Temagami BIF show a remarkable preservation and provides a unique geochemical archive for ancient seawater some 2.7 Ga ago.

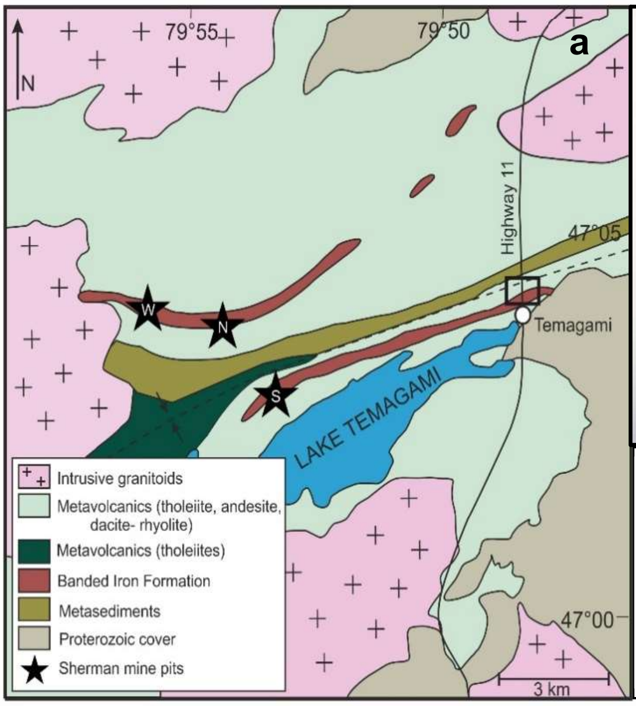

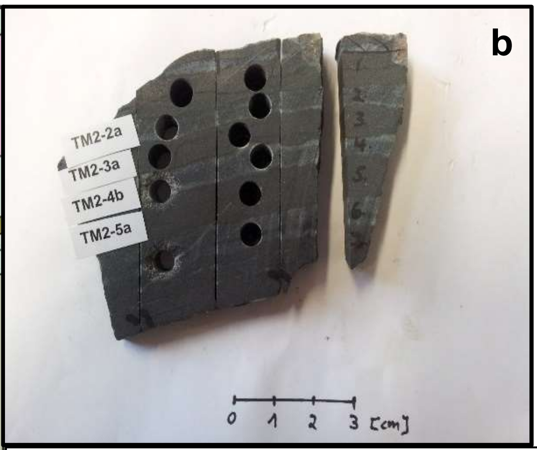

Supplementary Figure 5. **a)** Simplified geological map of the Temagami Greenstone Belt (simplified from Bowins & Crocket, 1994) showing the sampling location (black square) and positions of the Sherman Mine pits (W = west, N = north, S = south). **b)** Temagami BIF sample TM2 representative of all analyzed Temagami BIF samples in this study showing alternating (meta)chert and magnetite mesobands.

Sampling and intra-band W homogeneity

Individual Fe- and Si-rich layers corresponding to the Temagami BIF samples analyzed in prior studies[27,31] were selected for this study. While both mentioned studies used micro drill cores of individual layers (ca. 400 mg), the required amounts for high-precision ^{182}W analyses exceeded the amount gained by micro drill cores. Thus, parts of the individual layers macroscopically free of secondary veins and weathering rims were cut by a diamond-bearing saw and crushed in an agate mill to homogenous sample powders (ca. 1-3 g).

Direct major and trace element data of the sample splits analyzed for ^{182}W are not available. However, there is good evidence that W is homogeneously distributed within individual BIF layers. Two individual studies[27,31] analyzed different micro drill cores from the same BIF layer few centimeters apart from each other using two different high-temperature, high-pressure digestion methods. The results of both studies yield W concentration differences below 8 % for six out of nine samples with only two samples deviating in the order of 20-30 % and one sample with 52 % (Supplementary Table 1). The overall good agreement suggests that W concentrations are homogenously distributed between individual BIF layers and may be used to evaluate syn- and post-depositional alteration processes on the W budget of each Temagami BIF layer.

Layer	type	Bau & Alexander 2009	Viehmann et al. 2014, unpublished	difference in %
TM1-3a (FUM23)	Mgt	0.13	0.124	4.8
TM1-3b (FUM 25)		0.147	0.146	0.7
TM1-4	Mgt	0.222	0.284	21.8
TM2-2 (FUM26)	Chert	0.205	0.135	51.9
TM2-3 (FUM27)	Mgt	0.299	0.285	4.9
TM2-4 (FUM29)	Chert	0.161	0.221	27.1
TM2-5 (FUM28)	Mgt	0.377	0.365	3.3
TM3-3 (FUM30)	Chert	0.328	0.355	7.6
TM3-5 (FUM31)	Chert	0.61	0.628	2.9

Supplementary Table 1: Comparison of W concentration data from Bau & Alexander (2009) and Viehmann et al. (2014). Mgt – magnetite

Sample purity

The prerequisite to apply geochemical proxies to marine chemical sediments is the use of exceptionally pure sample material, i.e. samples whose geochemical budget is not masked by syn- or post-depositional processes. Two prior studies[27,31] already confirmed negligible effects of syn- (detrital contamination) and post-depositional alteration (diagenesis, metamorphism) on the geochemical composition of the exact similar BIF layers used in this study. However, a careful evaluation of detrital contamination and post-depositional processes on the W budget of the individual magnetite and (meta)chert layers of the 2.7 Ga old Temagami BIF is also given below:

Detrital contamination

Detrital contamination in marine chemical sediments such as BIFs or carbonates is commonly monitored by correlations between the element/isotope system of interest with almost immobile

elements that are clearly associated with detrital aluminosilicates (e.g., Al, Zr, Hf, Sc, Ti, etc.)[32,33]. Temagami shales (e.g., SMS-8, 0.374 ppm) have W concentrations within the range of the studied magnetite and (meta)chert layers (0.175 – 1.04 ppm). Thus, detrital contamination on the W budget has to be monitored carefully:

No correlation of W concentrations with Zr, an immobile element clearly associated with detrital aluminosilicates, in both Fe- and Si-rich layers can be observed, but also no correlation between these elements within layers of the same type (Supplementary Figure 4a). The weak correlation (r^2=0.3899) between Zr concentrations and $\mu^{182}W$ isotope composition of individual (meta)chert and magnetite layers may be recognized. However, this correlation likely results from minor amounts of (meta)chert and magnetite source mixing, rather than contamination by detrital components. If the W isotopic compositions of the chemical sediments were variably affected by detrital contamination then the BIF samples would be expected to plot on a two-component mixing curve between a detrital and a seawater endmember. However, this is not observed (Supplementary Figure 4b). In fact, as shown in Supplementary Figure 4b, the observed correlation does not fall on the mixing curve between the studied shales, most representative for detrital input from the Temagami Greenstone Belt, and sample TM3-7, the (meta)chert with the most positive $\mu^{182}W$ composition, most closely representing ancient seawater with the lowest immobile element concentrations.

In conclusion, W concentrations and ^{182}W isotopic compositions in the Temagami BIF have not been significantly affected by detrital contamination. The distinct $\mu^{182}W$ differences between magnetite and (meta)chert bands are rather dependent on mineralogy and, thus, truly reflect the W isotopic composition of water masses from which the chemical sediment precipitated some 2.7 Ga ago.

Post-depositional alteration

There are several combined arguments that the here studied BIF samples do not show post-depositional alteration of the pristine $\mu^{182}W$ isotopic composition representing the 182W composition of 2.7 Ga Temagami seawater:

1. Post-depositional alteration of the element and its isotopes of interest in marine chemical sediments is commonly monitored by correlations with fluid-mobile elements such as Sr. Positive correlations, i.e., increasing element concentrations of both elements are expected during mineralization processes, while negative correlations, i.e., decreasing elemental concentrations are expected during fluid-rock interactions that effectively leach the respective elements from the surrounding rocks. In the Temagami BIF samples, no correlation between W and Sr concentrations can be observed (r^2=0.0345; Supplementary Figure 4c). Similarly, $\mu^{182}W$ values of Fe- and Si-rich samples show no correlations with Sr (r^2=0.0312; Supplementary Figure 4d), indicating that the isotopic composition of the here analyzed rocks could not have been significantly affected by post-depositional processes.

2. Magnetite and (meta)chert samples show distinct bimodal $\mu^{182}W$ compositions. If post-depositional alteration processes had significantly affected the $\mu^{182}W$ isotope composition in the studied samples, then a homogenization of not only the concentrations but also the $\mu^{182}W$ signatures in both the Fe-rich and Si-rich bands would be expected. This, however, is not observed in the presented data (Table 1, Figure 1, main text).

Supplementary References

1. Touboul Mathieu, Puchtel Igor S., & Walker Richard J. 182W Evidence for Long-Term Preservation of Early Mantle Differentiation Products. *Science* **335**, 1065–1069 (2012).

2. Willbold, M., Elliott, T. & Moorbath, S. The tungsten isotopic composition of the Earth's mantle before the terminal bombardment. *Nature* **477**, 195–198 (2011).

3. Mundl, A. *et al.* Tungsten-182 heterogeneity in modern ocean island basalts. *Science* **356**, 66 (2017).

4. Mundl-Petermeier, A. *et al.* Temporal evolution of primordial tungsten-182 and 3He/4He signatures in the Iceland mantle plume. *Chem. Geol.* **525**, 245–259 (2019).

5. Mundl-Petermeier, A. *et al.* Anomalous 182W in high 3He/4He ocean island basalts: Fingerprints of Earth's core? *Geochim. Cosmochim. Acta* **271**, 194–211 (2020).

6. Rizo, H. *et al.* 182W evidence for core-mantle interaction in the source of mantle plumes. *Geochem. Perspect. Lett.* **11**, 6–11 (2019).

7. Tusch, J. *et al.* Convective isolation of Hadean mantle reservoirs through Archean time. *Proc. Natl. Acad. Sci.* **118**, e2012626118 (2021).

8. Mei, Q.-F., Yang, J.-H., Wang, Y.-F., Wang, H. & Peng, P. Tungsten isotopic constraints on homogenization of the Archean silicate Earth: Implications for the transition of tectonic regimes. *Cont. Orig. Evol. Interact. Reserv.* **278**, 51–64 (2020).

9. Reimink, J. R. *et al.* Tungsten Isotope Composition of Archean Crustal Reservoirs and Implications for Terrestrial µ182W Evolution. *Geochem. Geophys. Geosystems* **21**, e2020GC009155 (2020).

10. Mundl, A., Walker, R. J., Reimink, J. R., Rudnick, R. L. & Gaschnig, R. M. Tungsten-182 in the upper continental crust: Evidence from glacial diamictites. *Chem. Geol.* **494**, 144–152 (2018).

11. Kruijer, T. S. & Kleine, T. No 182W excess in the Ontong Java Plateau source. *Chem. Geol.* **485**, 24–31 (2018).

12. Puchtel, I. S., Blichert-Toft, J., Touboul, M. & Walker, R. J. 182W and HSE constraints from 2.7 Ga komatiites on the heterogeneous nature of the Archean mantle. *Geochim. Cosmochim. Acta* **228**, 1–26 (2018).

13. Puchtel, I. S., Blichert-Toft, J., Touboul, M., Horan, M. F. & Walker, R. J. The coupled 182W-142Nd record of early terrestrial mantle differentiation. *Geochem. Geophys. Geosystems* **17**, 2168–2193 (2016).

14. Tusch, J. *et al.* Uniform 182W isotope compositions in Eoarchean rocks from the Isua region, SW Greenland: The role of early silicate differentiation and missing late veneer. *Geochim. Cosmochim. Acta* **257**, 284–310 (2019).

15. Willbold, M., Mojzsis, S. J., Chen, H.-W. & Elliott, T. Tungsten isotope composition of the Acasta Gneiss Complex. *Earth Planet. Sci. Lett.* **419**, 168–177 (2015).

16. Touboul, M., Liu, J., O'Neil, J., Puchtel, I. S. & Walker, R. J. New insights into the Hadean mantle revealed by 182W and highly siderophile element abundances of supracrustal rocks from the Nuvvuagittuq Greenstone Belt, Quebec, Canada. *Chem. Geol.* **383**, 63–75 (2014).

17. Liu, J., Touboul, M., Ishikawa, A., Walker, R. J. & Graham Pearson, D. Widespread tungsten isotope anomalies and W mobility in crustal and mantle rocks of the Eoarchean Saglek Block, northern Labrador, Canada: Implications for early Earth processes and W recycling. *Earth Planet. Sci. Lett.* **448**, 13–23 (2016).

18. Puchtel, I. S. *et al.* Lithophile and siderophile element systematics of Earth's mantle at the Archean–Proterozoic boundary: Evidence from 2.4Gakomatiites. *Geochim. Cosmochim. Acta* **180**, 227–255 (2016).

19. Puchtel, I. S. *et al.* Ultra-depleted 2.05 Ga komatiites of Finnish Lapland: Products of grainy late accretion or core-mantle interaction? *Chem. Geol.* **554**, 119801 (2020).

20. Rizo, H. *et al.* Early Earth differentiation investigated through 142Nd, 182W, and highly siderophile element abundances in samples from Isua, Greenland. *Geochim. Cosmochim. Acta* **175**, 319–336 (2016).

21. Dale, C. W., Kruijer, T. S. & Burton, K. W. Highly siderophile element and 182W evidence for a partial late veneer in the source of 3.8 Ga rocks from Isua, Greenland. *Earth Planet. Sci. Lett.* **458**, 394–404 (2017).

22. Reimink, J. R. *et al.* Petrogenesis and tectonics of the Acasta Gneiss Complex derived from integrated petrology and 142Nd and 182W extinct nuclide-geochemistry. *Earth Planet. Sci. Lett.* **494**, 12–22 (2018).

23. Archer, G. J. *et al.* Lack of late-accreted material as the origin of 182W excesses in the Archean mantle: Evidence from the Pilbara Craton, Western Australia. *Earth Planet. Sci. Lett.* **528**, 115841 (2019).

24. Tappe, S., Budde, G., Stracke, A., Wilson, A. & Kleine, T. The tungsten-182 record of kimberlites above the African superplume: Exploring links to the core-mantle boundary. *Earth Planet. Sci. Lett.* **547**, 116473 (2020).

25. Nakanishi, N. *et al.* Tungsten-182 evidence for an ancient kimberlite source. *Proc. Natl. Acad. Sci.* **118**, e2020680118 (2021).

26. Peters, B. J., Mundl-Petermeier, A., Carlson, R. W., Walker, R. J. & Day, J. M. D. Combined Lithophile-Siderophile Isotopic Constraints on Hadean Processes Preserved in Ocean Island Basalt Sources. *Geochem. Geophys. Geosystems* **22**, e2020GC009479 (2021).

27. Bau, M. & Alexander, B. W. Distribution of high field strength elements (Y, Zr, REE, Hf, Ta, Th, U) in adjacent magnetite and chert bands and in reference standards FeR-3 and FeR-4 from the Temagami iron-formation, Canada, and the redox level of the Neoarchean ocean. *Precambrian Res.* **174**, 337–346 (2009).

28. Fyon, J. A. & Cole, S. Geology of part of the Temagami greenstone belt, District of Nipissing, including relationships between lithological, alteration, and structural features and precious-metal occurrences, in Summary of fieldwork and other activities 1989. *Ont. Geol. Surv. Misc. Pap.* **146**, 108–115 (1989).

29. Bowins, R. J. & Crocket, J. H. Sulfur and carbon isotopes in Archean banded iron formations: Implications for sulfur sources. *Chem. Geol.* **111**, 307–323 (1994).

30. Bowins, R. J. & Heaman, L. M. Age and timing of igneous activity in the Temagami greenstone belt, Ontario: a preliminary report. *Can. J. Earth Sci.* **28**, 1873–1876 (1991).

31. Viehmann, S., Hoffmann, J. E., Münker, C. & Bau, M. Decoupled Hf-Nd isotopes in Neoarchean seawater reveal weathering of emerged continents. *Geology* **42**, 115–118 (2014).

32. Bau, M. Effects of syn- and post-depositional processes on the rare-earth element distribution in Precambrian iron-formations. *Eur. J. Mineral.* **5**, 257–268 (1993).

33. Viehmann, S. *et al.* The reliability of ~2.9 Ga old Witwatersrand banded iron formations (South Africa) as archives for Mesoarchean seawater: Evidence from REE and Nd isotope systematics. *J. Afr. Earth Sci.* **111**, 322–334 (2015).
