# Supplementary material for: Earth’s geodynamic evolution constrained by 182W in Archean seawater
Source: Nat Commun. 2022 May 16;13:2701. doi: 10.1038/s41467-022-30423-3 (PMC9110358; doi:10.1038/s41467-022-30423-3)
Supplement: Supplementary file 2 — Description of Additional Supplementary Files [file 41467_2022_30423_MOESM2_ESM.pdf]

Description of Additional Supplementary Files

Supplementary Data 1. All data obtained using thermal ionization mass spectrometry in negative ionization mode at the University of Vienna. Measured $^{182}W/^{184}W$ normalized to $^{186}W/^{183}W$ and $^{186}W/^{184}W$ and $^{183}W/^{184}W$ normalized to $^{186}W/^{184}W$ ratios as well as calculated µ-notations for each analytical session. Data table includes all analysed standard solutions and samples.

Supplementary Data 2. All data obtained using multi-collector inductively coupled plasma mass spectrometry at the University of Cologne. Data table includes individual measurements (calculated µ-notations) of all samples and in-house reference materials.
